# Comment On: Piccolo Device for Treating Patent Ductus Arteriosus Beyond Premature Newborn Patients, a Novel Experience in South America: A Retrospective Study

**DOI:** 10.1002/hsr2.71725

**Published:** 2026-01-04

**Authors:** Asad Ali, Rayyan Ahmad Zaka

**Affiliations:** ^1^ Department of Medicine Sheikh Khalifa Bin Zayed Al Nayhan Medical and Dental College Lahore Punjab Pakistan

To the editor,

We read with great interest the research paper by Aristizabal et al. [1] on the treatment of Patent Ductus Arteriosus (PDA) using the Piccolo Device, which is a retrospective study. We would like to applaud Aristizabal et al for their efforts in highlighting this novel experience. This piece beautifully discusses the utility of the Piccolo device for treating PDA in patients beyond the neonatal phase, precisely demonstrating its efficacy and safety in older geriatric patients, without a significant increase in complications or mortality rates. While these findings provide valuable insights, several limitations, some of which are mentioned by the author, as well as others not mentioned, warrant discussion. We would like to highlight some key methodological limitations that may influence the generalizability of the results. The study employs a retrospective design in a single center, which limits its external validity and introduces potential selection bias. In contrast, Sathanadam et al. [2] conducted a prospective, multicenter trial enrolling 200 patients across nine centers, ensuring broader applicability. The small sample size (*n* = 29) in the study under discussion restricts statistical power, particularly in detecting rare follow‐up complications.

In contrast, Morray et al. [3] addressed this by enrolling 200 patients across three centers, thereby improving both statistical validity and representativeness. Moreover, limited and variable follow‐up (mean, 8 months) may underestimate the incidence of late complications. In comparison, Morray et al. [3] conducted a structured 3‐year follow‐up to assess residual shunts and adverse events carefully. The discussion compares the results to those of extensive multicentre studies and concludes that they are comparable or equally effective. Future studies should aim for prospective multi‐center designs with larger cohorts and uniform follow‐up protocols, as seen in Baspinar et al. [4]. Outcomes were discussed as a whole. Still, no subgroup analysis was conducted (i.e., > 2 kg vs. < 2 kg), as in Sathanandam et al. [2], because the two groups may vary in terms of complications and results. Sub‐group comparisons should be encouraged in future studies. The discussion overlooks the operator's learning curve, which may significantly impact the outcome, particularly in a single‐center study. An operator's skill and device familiarity can influence the results, as evidenced in past studies [5]; thus, a brief note should have discussed this.

We appreciate the author's effort and hope our comments added value to the discussion.

## Author Contributions

**Asad Ali:** conceptualization, methodology, writing – review and editing, writing – original draft, project administration, supervision. **Rayyan Ahmad Zaka:** writing – review and editing, validation, writing – original draft.

## Funding

The authors received no specific funding for this work.

## Ethics Statement

Ethical approval is not required for this study because it is a letter to the editor based on publicly available data and does not involve human or animal subjects.

## Consent

The authors have nothing to report.

## Conflicts of Interest

The authors declare no conflicts of interest. All authors have read and approved the final version of the manuscript. Corresponding Asad Ali had full access to all of the data in this study and takes full responsibility for the integrity of the data and the accuracy of data analysis.

## Declaration of Generative AI and AI‐Assisted Technologies in the Writing Process

During the preparation of this work, the authors used OpenAI's ChatGPT in order to improve the written manuscript. After using the tool/service, the authors reviewed and edited the content as needed and take full responsibility for the content of the publication.

## Transparency Statement

The lead author Asad Ali affirms that the manuscript is an honest, accurate, and transparent account of the study being reported; that no important aspects of the study have been omitted; and that any discrepancies from the study planned (and, if relevant, registered) have been explained.

## Data Availability

Data sharing is not applicable to this article as no new data were created or analyzed in this study.
